# Background frequencies for residue variability estimates: BLOSUM revisited

**DOI:** 10.1186/1471-2105-8-488

**Published:** 2007-12-27

**Authors:** I Mihalek, I Reš, O Lichtarge

**Affiliations:** 1 Department of Molecular and Human Genetics, Baylor College of Medicine, One Baylor Plaza, Houston, TX 77030, USA; 2 Bioinformatics Institute, Agency for Science, Technology and Research (A*STAR), 30 Biopolis Street, #07-01 Matrix, 138671, Singapore

## Abstract

**Background:**

Shannon entropy applied to columns of multiple sequence alignments as a score of residue conservation has proven one of the most fruitful ideas in bioinformatics. This straightforward and intuitively appealing measure clearly shows the regions of a protein under increased evolutionary pressure, highlighting their functional importance. The inability of the column entropy to differentiate between residue types, however, limits its resolution power.

**Results:**

In this work we suggest generalizing Shannon's expression to a function with similar mathematical properties, that, at the same time, includes observed propensities of residue types to mutate to each other. To do that, we revisit the original construction of BLOSUM matrices, and re-interpret them as mutation probability matrices. These probabilities are then used as background frequencies in the revised residue conservation measure.

**Conclusion:**

We show that joint entropy with BLOSUM-proportional probabilities as a reference distribution enables detection of protein functional sites comparable in quality to a time-costly maximum-likelihood evolution simulation method (rate4site), and offers greater resolution than the Shannon entropy alone, in particular in the cases when the available sequences are of narrow evolutionary scope.

## Background

As a groundwork for the mutational study of a protein, many researchers will choose the comparative analysis of the protein homologues. Column entropy in the multiple sequence alignment [1,2] has proven over time as a workhorse of such endeavors, giving an excellent estimate of residue variability, and proving difficult to beat in terms of its prediction power. One of its limitations, which we address in this paper, is its inability to differentiate between amino acid residue types. For example, its straightforward application proves blind to the fact that an isoleucine, a residue of a type that mutates easily, when found conserved over a large evolutionary distance, should appear more conspicuous than a conserved proline. Shannon's entropy is unable to distinguish between the two cases, and thus its resolution stops at the level of residues which are completely conserved across the aligned homologue set.

**Figure 1:**
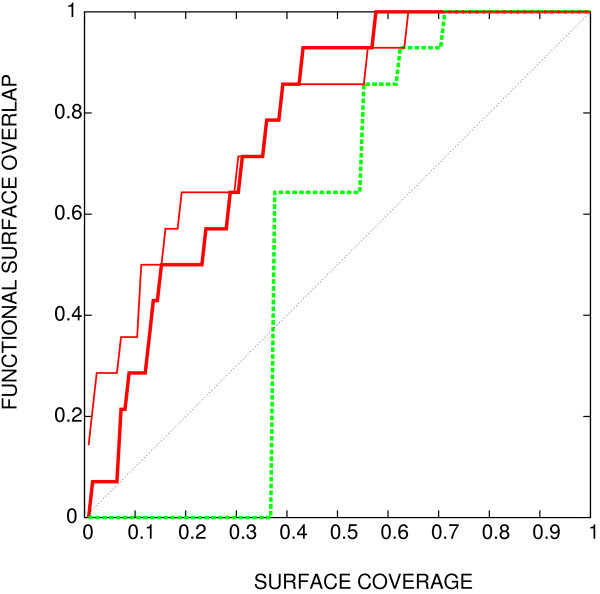
**Methods comparison, using rabphilin-3a as an example**. The ability of three different methods to detect the interacting surface of the small G protein rab3a with the effector domain of rabphilin-3a (PDB [27] identifier 1zbd, chain A). Horizontal axis: fraction of surface appearing among the top scoring residues (surface coverage). Vertical axis: fraction of interface detected. Thin red line: rate4site; green dashed line: column entropy; thick red line: joint entropy with BLOSUM background frequency, described in the text.

This is illustrated in Figure 1 where entropy (green dashed line) is compared with a prediction from a detailed simulation of evolutionary events, provided by rate4site program [3] (red thick full line; the thin line gives a preview of the method described in this work). The most prominent feature of the simulation result is that the simulation can differentiate among the 35% of residues which are invariant in this alignment. This capability can be traced back to the fact that the mutation rates used in the simulation are residue type-dependent – a distinguishing capability that Shannon's entropy lacks. This shortcoming makes application of Shannon's entropy particularly awkward in the cases where the available homologues are few and closely related to the query.

While the entropy is by no means the only method to estimate residue conservation ([4-7] and references therein), and is not suitable for the identification of functional determinants [8], we leave it as a focus of our attention for its central role in the existing research (for example, [9]), and its potential as a building block for more elaborate methods [10].

In order to introduce mutational preferences of different residue types into the analysis, we turn to joint entropy with Kullback-Leibler-like [11] background frequencies. The joint entropy allows consideration of mutational events in terms of residue pairs (*x*_1_, *x*_2_) (for mutation from *x*_1 _to *x*_2_, or vice versa), whereas the background frequencies enable the estimate of the statistical (im)probability of an observed mutation occurring at random. The background frequencies, we suggest, are already available in terms of BLOSUM matrices, even though some adjusting is needed to turn them into matrices of transition probabilities. In distinction from earlier works using joint entropy with Kullback-Leibler background distribution to detect co-evolution across multiple alignment columns (e.g [12]), we propose, closer in spirit to BLOSUM itself, considering joint entropy for a single alignment column (a "sum-of-pairs" type of score [4]). To establish the reasonableness of the approach, we first argue that the expression for joint entropy, when applied to a single distribution, has the properties of entropy in the general Shannon sense, but at the same time allows introduction of a phenomenological (Kullback-Leibler [11]) term describing the difference in residue types and in their mutational preferences. We then turn the raw set of data, from which the BLOSUM matrices were derived, into a mutation probability matrix, and then apply the resulting formula to the detection of a set of protein interfaces. The method shows a significant improvement in the specificity of detection of functional surfaces starting from a small set of close homologues, as illustrated on a test set of 18 transiently interacting homodimers.

## Method

A column in a multiple sequence alignment can be thought of in the following way: If the sequence set were a fair sample of all possible orthologs, and the variability of each residue depended only on its type, the amino acid population in each column would reflect the ease with which they are exchangeable in a general case. Setting aside the problem of the fairness of sample, which we do not attempt to address here, the difference from the expected distribution is a result of the particular evolutionary forces on the residue, or the lack thereof.

The Shannon entropy of an alignment column – represented by a distribution of residue types *X *– is evaluated as

$$H\left(X\right)=-\sum_{x}P\left(x\right)lnP\left(x\right),$$

where *x *is one of 20 residue types, and the probability of occurrence of *x*, *P*(*x*), is estimated by *f*(*x*), the frequency of the appearance of residue type within the alignment column:

$$P(x)\approx f(x)=\frac{N(x)}{L},$$

where *N*(*x*) is the number of appearances of residue type *x*, and *L *is the length of the column. To find an expression which will incorporate residue mutation propensity, we first look at the expression for joint entropy of two distributions

$$H(X,Y)=-\sum_{x}\sum_{y}P(x,y)\ln P(x,y),$$

and apply it to a single distribution, *X*:

$$H(X,X)=-\sum_{x_{1}}\sum_{x_{2}\leq x_{1}}P(x_{1},x_{2})\ln P(x_{1},x_{2}).$$

**Figure 2:**
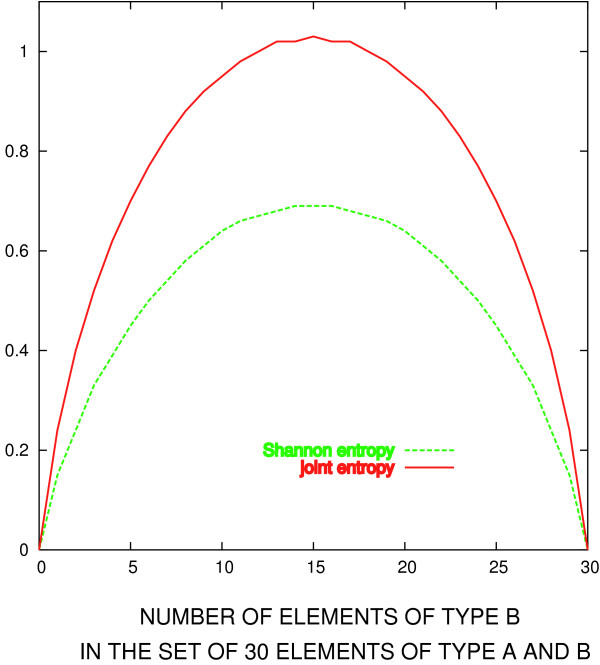
**Shannon and joint entropies**. Comparison between Shannon and joint entropies, in the hypothetical case of a set of 30 elements of type A or B. Red line: joint entropy, evaluated according to Eq. 4. Green line: Shannon entropy (Eq. 3).

*P*(*x*_1_, *x*_2_) is now estimated by the frequency of residue type pairs which can be formed from the residues in the column:

$$P(x_{1},x_{2})\approx \frac{N(x_{1},x_{2})}{L(L-1)/2}$$

where *N*(*x*_1_, *x*_2_) is the number of unordered pairs (*x*_1_, *x*_2_), which can be formed by taking both *x*_1 _and *x*_2 _from the distribution *X*, and *L *is the column length. The quantity *P*(*x*_1_, *x*_2_) behaves the same way as the Shannon entropy, as illustrated in Figure 2, for the case of a set of 30 elements of types *A *and *B*. This corresponds to a case of a column taken from an alignment of 30 sequences, and which happens to contain only two residue types. Just as in the case of Shannon's entropy (dashed line), the entropy function defined in Eq. 4 is zero when the set contains only one type of element (i.e. only one residue type), and maximal when the two types are equally represented.

The joint entropy also has the advantage that it allows for easy incorporation of information about mutational preference of amino acids, following the approach of Kullback and Leibler:

$$H_{BB}(X,X)=-\sum_{x_{1}}\sum_{x_{2}\leq x_{1}}P(x_{1},x_{2})\ln\frac{P(x_{1},x_{2})}{Q(x_{1},x_{2})}.$$

*Q*(*x*_1_, *x*_2_) here plays the role of the "background" mutation propensity. In particular, *P*(*x*_1_, *x*_1_) which is greater than *Q*(*x*_1_, *x*_1_) will result in negative *H*_*BB*_, indicating that the residue is more conserved than its average mutation propensity would dictate (see also the example below). The most conserved residue still has the minimal score (as in the case of Shannon entropy) which can in this case be less than zero. To estimate *Q*, we take a matrix of raw pair frequencies originally assembled for the calculation of BLOSUM matrices [13,14]. These matrices are not probabilities, but counts of pairs of different amino acid types appearing in the same alignment column. Thus, we first normalize each row to unity. The distribution *P *described in Eq. 5 and used in Eq. 6 has no way of distinguishing between the two possible orderings of its arguments; that is, in this model we do not know which residue type was "earlier" and which one was "later" – mutations in both direction are equally probable (for a model aimed at capturing the difference, see [15]). Therefore, we need the reference distribution *Q *which possesses the same symmetry. The matrix obtained by normalizing the rows in the raw BLOSUM table is no longer symmetrical, so we approximate it with a nearest (in terms of the average root-mean-square distance between the elements) symmetric matrix whose rows and columns sum up to 1. To find *Q *we use a Monte Carlo procedure: starting from 20 × 20 identity matrix, we subtract (add) a small quantity from a randomly chosen off-diagonal element, and add (subtract) it from the two corresponding diagonal elements.

The optimized (minimized, in this case) quantity is root-mean-square distance of matrix elements to the starting (BLOSUM frequency) matrix. The *Q *matrix used in this work was derived from the frequencies in 35% clustering blocks, and can be found in Additional file 1.

To illustrate the way *H*_*BB *_scores residue columns, we look at two simple examples. First we compare the scoring of two completely conserved columns, one with isoleucines, and one with prolines:

$$\begin{array}{cc} \text{I} & \text{P}\\ \text{I} & \text{P}\\ \text{I} & \text{P}\\ \text{I} & \text{P} \end{array}$$

Since *Q*(*I*, *I*) = 0.14, and *Q*(*P*, *P*) = 0.29 (see Additional file 1; *P*(*x*_1_, *x*_2_) is equal to 1 for any conserved column), the value of *H*_*BB *_for the first column is -1.9, and for the second -1.2. Remembering that, just as in the case of Shannon column entropy, the lower number indicates higher degree of conservation, the isoleucine column is by this reasoning under higher evolutionary pressure than proline. That is, since isoleucine is quite prone to mutation (to a valine, for example), we find it as an element of surprise that it is completely conserved, and attribute this to a special role alanine plays at this particular position in the protein.

In a slightly more complex example we compare two columns with two values of amino acid types each:

$$\begin{array}{cc} \text{I} & \text{I}\\ \text{I} & \text{I}\\ \text{I} & \text{I}\\ \text{V} & \text{P} \end{array}$$

Perhaps counterintuitively, the second column scores better (*H*_*BB *_= -1.4, compared with *H*_*BB *_= -1.1 for the first column), largely because of the contribution of *Q*(*I*, *P*) = 0.04 (as opposed to common substitution *I *↔ *V *with *Q*(*I*, *V*) = 0.12). If it is true that in the evolutionary history of our hypothetical protein the isoleucine at this position was replaced by a proline, then this position must be very special, claims this model, perhaps conferring specificity to the proteins function. (As a corollary, the whole process depends critically on reliability of the alignment. We therefore expect this approach to become problematic for very distant homologues, as the alignments become unreliable – a common problem in comparative analysis of proteins.)

### The test set

The test set used here consists of 18 transiently interacting heterodimers, a subset of the set originally published by Nooren and Thornton [16,17], resulting in 36 interfaces. Out of 36 protomers in this set, 10 are classified as all *a*-helix in the SCOP [18] scheme, 5 as all *β*-sheet, 15 as *α*/*β*, 4 as *α *+ *β*, and 2 simply as "small proteins" (see Additional file 1). The interface residues are defined as the set of residues which upon complexation become completely isolated from the water molecules, or can be found in the vicinity of coordinated water molecule(s). Such regions are either in close contact with the interacting partner, enabling short range interactions (perhaps mediated by coordinated waters), or functioning as hydrophobic "suction pumps;" in either case they are expected to be responsible for the interaction strength and specificity, and thus under increased evolutionary pressure. [17]

The HSSP [19] alignment was used as the initial alignment in all cases presented. Sequences aligning with less than 75% of the query length were removed from the alignment. For each pair of sequences more than 98% identical, the shorter sequence was discarded. If the average identity of any two sequences, measured by an average over all windows 20 residues long, was below 50%, the sequence with the smaller percent identity to the query was discarded. In the same way, all sequences were required to have at least 70% identity. These strict requirements were used precisely to illustrate the point that the presented method can extract interesting information even from very closely related sequences.

For comparison, the results are also shown for the same set of proteins, but using a set of more distant homologues for each family – sequences at least 15% identical to the query and among themselves. In some cases (1a0oF, 1c1yB, 1ceeB, 1cxzB, 1foeA, 1he1A, 1lfdA, 1rrpA, 1wq1G, 1zbdB and 2trcP) this procedure – using the HSSP alignment as a starting point – still resulted in a set of very similar homologues. In these cases we resorted to 4 iterations of PSIBlast [20] search on the NCBI non-redundant database of protein sequences [21], with the cutoff E-value of 0.05, followed by alignment using Muscle [22], and pruning of (dis)similar sequences as described above for the HSSP case.

## Results and discussion

**Figure 3:**
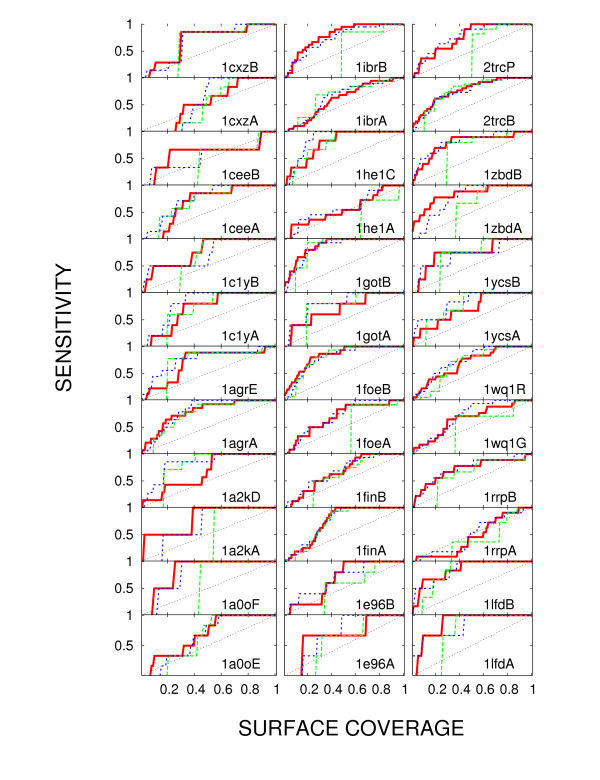
**Testing the performance of *H*_*BB *_on several protein-protein interfaces**. Sensitivity as a function of surface coverage for alignments of close homologues. Protein Databank Identifier of each protein is indicated in the corner of each panel. The results are based on sets of homologues very close to each query. In each case, all methods were applied to the same alignment. Full red line: joint entropy, evaluated according to Eq. 4; dashed green line: Shannon entropy; dashed blue line: rate4site.

**Figure 4:**
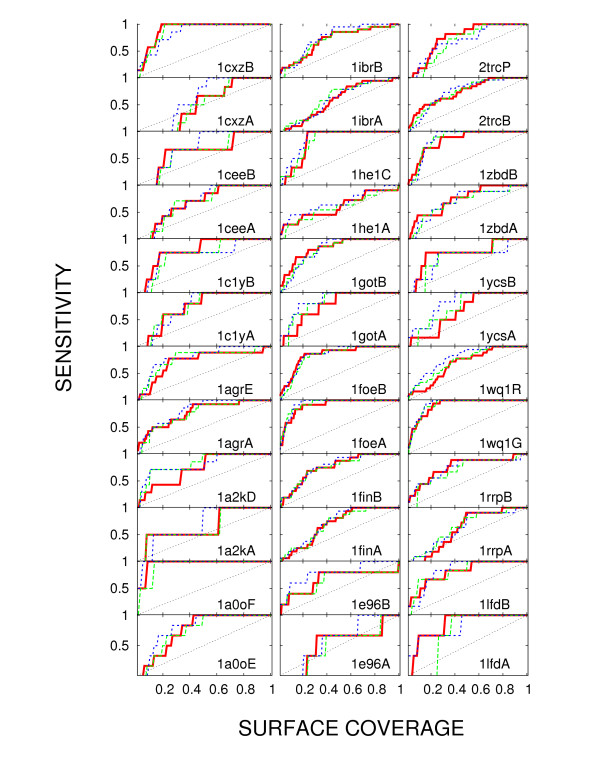
**Testing the performance of *H*_*BB*_: broad evolutionary sample**. The same as Figure 3, using alignments encompassing more distant homologues.

Figures 3 and 4 show the performance of *H*_*BB *_(Eq. 6; red) in detecting protein interface, compared with the column entropy (green) and rate4site (blue). The results are presented in terms of sensitivity versus surface coverage curves. Definitions of sensitivity and coverage stem from our use of methods which, in one way or another, rank residues by the evolutionary pressure they experience. *Coverage *in this context refers to the fractional overlap of certain percentage of top ranking residues with the set of surface residues, while *sensitivity *is the overlap of the same top ranking residues with the target set of interface residues. The question of the optimal choice of coverage (or of the underlying *H*_*BB *_value) is left open, with the understanding that a higher coverage choice detects a larger number of test residues, but also leads to a larger number of false positives. The quality of any method consists precisely of its ability to maximize this hit-to-miss ratio.

The results in Figure 3 refer to a hypothetical and especially stringent case, in which only very close homologues (no less than 70% identity to the query) are available for the analysis. For the proteins in our test set this is not necessarily the case, but we limit the sequence selection to close homologues to illustrate our claim that *H*_*BB *_is then still able to extract information beyond the reach of Shannon's entropy. As shown in Figure 3*H*_*BB *_is capable of detecting parts of the interface down to one percent coverage of the entire surface, even using an evolutionarily narrow selection of sequences. At the same time, the results are quite comparable to the results obtained using a full-blown simulation of evolutionary events (rate4site; thin line). Taking the area under the sensitivity vs. coverage curve as an indicator of the prediction quality (the value of 1 is the maximal attainable), in a Wilcoxon signed-rank test [23], the areas resulting from the use of *H*_*BB *_are indeed different from those using entropy with the *p*-value *<*6 × 10^-5^. Using the same test, the quality of the predictions by *H*_*BB *_and rate4site are statistically indistinguishable (*p*-value of 0.4). Rate4site and *H*_*BB *_average the area of 0.73 and 0.72 respectively for this selection of sequences, while the entropy averages 0.62, thus indicating that both *H*_*BB *_and rate4site move the prediction toward more reliable. The last result is the consequence mostly of the inability of the entropy to achieve resolution at small coverage, thus decreasing the area under the curve.

In the following figure, Figure 4 we note that for a broader evolutionary coverage (sequences at least 15% identical to the query and among themselves), entropy becomes competitive again. However, *H*_*BB *_still performs comparably to rate4site, and even somewhat better than the column entropy. The average areas under the sensitivity-coverage curve are 0.74, 0.73, and 0.77 for *H*_*BB*_, entropy and rate4site respectively. On the Wilcoxon test, in this case of a sequence sample with lower homology, the results by *H*_*BB *_are more similar to those produced by column entropy (*p*-value 0.5) than by rate4site (*p*-value 0.01).

Information analogous to Figures 3 and 4, using Matthews correlation coefficient, is presented in Additional file 1: the success of the method varies from case to case, but it achieves the values of Matthews coefficient of up to 0.5.

The usefulness of the method is not limited to protein interfaces – it works as well as rate4site, and better than entropy in detection of catalytic sites for enzymes (see Additional file 1).

The model behind this approach acknowledges that starting from the the alignment column alone it is not possible to establish the residue type in the ancestral allele. Instead, the reasoning goes, in the lack of evolutionary pressure, the observed distribution should reflect the statistical propensity of residues to mutate to each other: if a residue type A is just as likely to mutate to type B as not to mutate at all, and vice versa, we expect to find the two types equally represented in a fair sample of existing alleles. A deviation from the uniform distribution, then, points to an external pressure to maintain a particular type, calling attention to the corresponding position in the protein sequence. This interpretation of the model makes its pitfalls obvious: a sequence sample produced automatically from currently available protein sequence databases is highly unlikely to be fair. (Valdar's method [4,24], for example, deals with the problem of fairness of sampling. For comparison, see Additional file 1.) Also, even though it tolerates small evolutionary breadth, since the method is inherently statistical, it requires a sizable number of sequences, a requirement shared with Shannon's entropy, but not with maximum likelihood methods (such as rate4site). Finally, and this is the problem common to all three methods discussed here, the pressure to conserve a particular physicochemical characteristic (such as acidity or aromaticity) goes undetected by *H*_*BB*. _However, with all of its shortcomings, the model immediately proves to be more useful (at least in the case of limited homology span) than the one oblivious to amino acid type, as indicated in Figure 3.

Consideration of the inherent problems may yet lead us to an improved approach.

## Conclusion

We have shown that a simple heuristic modification of Shannon entropy can match the prediction power of an elaborate evolution simulation. It is worth noting the advantages this brings: *H*_*BB *_is simple, which makes it applicable as a part of a more complex approach [10], and its speed (calculating a column score is several orders of magnitude faster than performing a simulation) makes it useful in repetitive tasks, such as optimization schemes [25]. In practical applications, the presented method can tackle much larger alignments, in terms of both number of sequences and their length, than an evolutionary simulation; in the opposite extreme (and contrary to the case of Shannon's entropy), the presented method can extract information from a very narrow evolutionary sample.

The data set used in this work is available at the Lichtarge Lab website [26].

## Authors' contributions

IM conceived of the study and implemented necessary software. The method was developed and the manuscript written through collaborative work of all authors. All authors read and approved the final manuscript.

## Supplementary Material

Additional file 1 Background frequencies for residue variability estimates: BLOSUM revisited – Supplementary Material. The supplement contains the reference distribution *Q*, structural classification of used proteins according to SCOP, and additional comparative analysis of the method presented here with methods already available in the literature. Click here for file
