# Supplementary material for: Background frequencies for residue variability estimates: BLOSUM revisited
Source: BMC Bioinformatics. 2007 Dec 27;8:488. doi: 10.1186/1471-2105-8-488 (PMC2267808; doi:10.1186/1471-2105-8-488)
Supplement: Additional file 1 — Background frequencies for residue variability estimates: BLOSUM revisited – Supplementary Material. The supplement contains the reference distribution Q, structural classification of used proteins according to SCOP, and additional comparative analysis of the method presented here with methods already available in the literature. [file 1471-2105-8-488-S1.pdf]

Background frequencies for residue variability estimates: BLOSUM revisited – Supplementary Material

I. Mihalek, I. Reš, and O. Lichtarge
Department of Molecular and Human Genetics,
Baylor College of Medicine, One Baylor Plaza, Houston, TX 77030

November 9, 2007

A	R	N	D	C	Q	E	G	H	I	L	K	M	F	P	S	T	W	Y	V
0.1681																			
0.0451	0.1917																		
0.0402	0.0376	0.1401																	
0.0467	0.0455	0.0704	0.2289																
0.0222	0.0208	0.0282	0.0178	0.3769															
0.0340	0.0530	0.0469	0.0326	0.0195	0.1177														
0.0527	0.0521	0.0516	0.0813	0.0529	0.0940	0.1696													
0.0848	0.0536	0.0931	0.0576	0.0444	0.0600	0.0476	0.2842												
0.0201	0.0253	0.0392	0.0254	0.0333	0.0257	0.0232	0.0201	0.2344											
0.0466	0.0357	0.0493	0.0324	0.0293	0.0369	0.0303	0.0263	0.0352	0.1416										
0.0626	0.0632	0.0565	0.0593	0.0594	0.0622	0.0069	0.0011	0.0640	0.1208	0.2021									
0.0585	0.0857	0.0636	0.0564	0.0458	0.0650	0.0738	0.0351	0.0441	0.0441	0.0195	0.1443								
0.0221	0.0241	0.0275	0.0130	0.0180	0.0310	0.0151	0.0178	0.0731	0.0273	0.0345	0.0234	0.0612							
0.0317	0.0375	0.0368	0.0253	0.0261	0.0325	0.0260	0.0250	0.0454	0.0500	0.0586	0.0361	0.0596	0.1903						
0.0359	0.0374	0.0350	0.0398	0.0250	0.0467	0.0461	0.0372	0.0552	0.0388	0.0288	0.0464	0.0289	0.0251	0.2932					
0.0719	0.0547	0.0587	0.0527	0.0377	0.0671	0.0542	0.0385	0.0554	0.0462	0.0037	0.0573	0.0490	0.0481	0.0450	0.1323				
0.0514	0.0375	0.0489	0.0401	0.0453	0.0458	0.0450	0.0348	0.0458	0.0452	0.0457	0.0495	0.0503	0.0417	0.0509	0.0677	0.1167			
0.0106	0.0135	0.0079	0.0079	0.0299	0.0394	0.0109	0.0101	0.0426	0.0089	0.0121	0.0123	0.3051	0.0463	0.0184	0.0097	0.0348	0.1789		
0.0313	0.0336	0.0274	0.0240	0.0267	0.0514	0.0274	0.0273	0.0592	0.0378	0.0379	0.0300	0.0477	0.0901	0.0255	0.0312	0.0281	0.1588	0.1440	
0.0633	0.0524	0.0411	0.0430	0.0409	0.0388	0.0396	0.0015	0.0333	0.1173	0.0012	0.0095	0.0714	0.0678	0.0407	0.0189	0.0746	0.0420	0.0608	0.1419

Table 1: Reference distribution $Q(x_1, x_2)$.

PDB chain	structural content according to SCOP
1agrE	All alpha
1agrA	All alpha
1a2kD	Alpha and beta (a/b)
1a2kA	Alpha and beta (a+b)
1a0oF	Alpha and beta (a+b)
1a0oE	Alpha and beta (a/b)
1cxzB	All alpha
1cxzA	Alpha and beta (a/b)
1ceeB	Small proteins
1ceeA	Alpha and beta (a/b)
1c1yB	Alpha and beta (a+b)
1c1yA	Alpha and beta (a/b)
1e96B	All alpha
1e96A	Alpha and beta (a/b)
1foeB	Alpha and beta (a/b)
1foeA	All alpha
1finB	All alpha
1finA	Alpha and beta (a+b)
1gotB	All beta
1gotA	All alpha
1he1C	Alpha and beta (a/b)
1he1A	All alpha
1ibrB	All alpha
1ibrA	Alpha and beta (a/b)
1lfdB	Alpha and beta (a/b)
1lfdA	Alpha and beta (a/b)
1rrpB	All beta
1rrpA	Alpha and beta (a/b)
1wq1R	Alpha and beta (a/b)
1wq1G	All alpha
1ycsB	All beta
1ycsA	All beta
1zbdB	Small proteins
1zbdA	Alpha and beta (a/b)
2trcP	Alpha and beta (a/b)
2trcB	All beta

Table 2: SCOP classification of the transient dimers used as the test set in the main text.

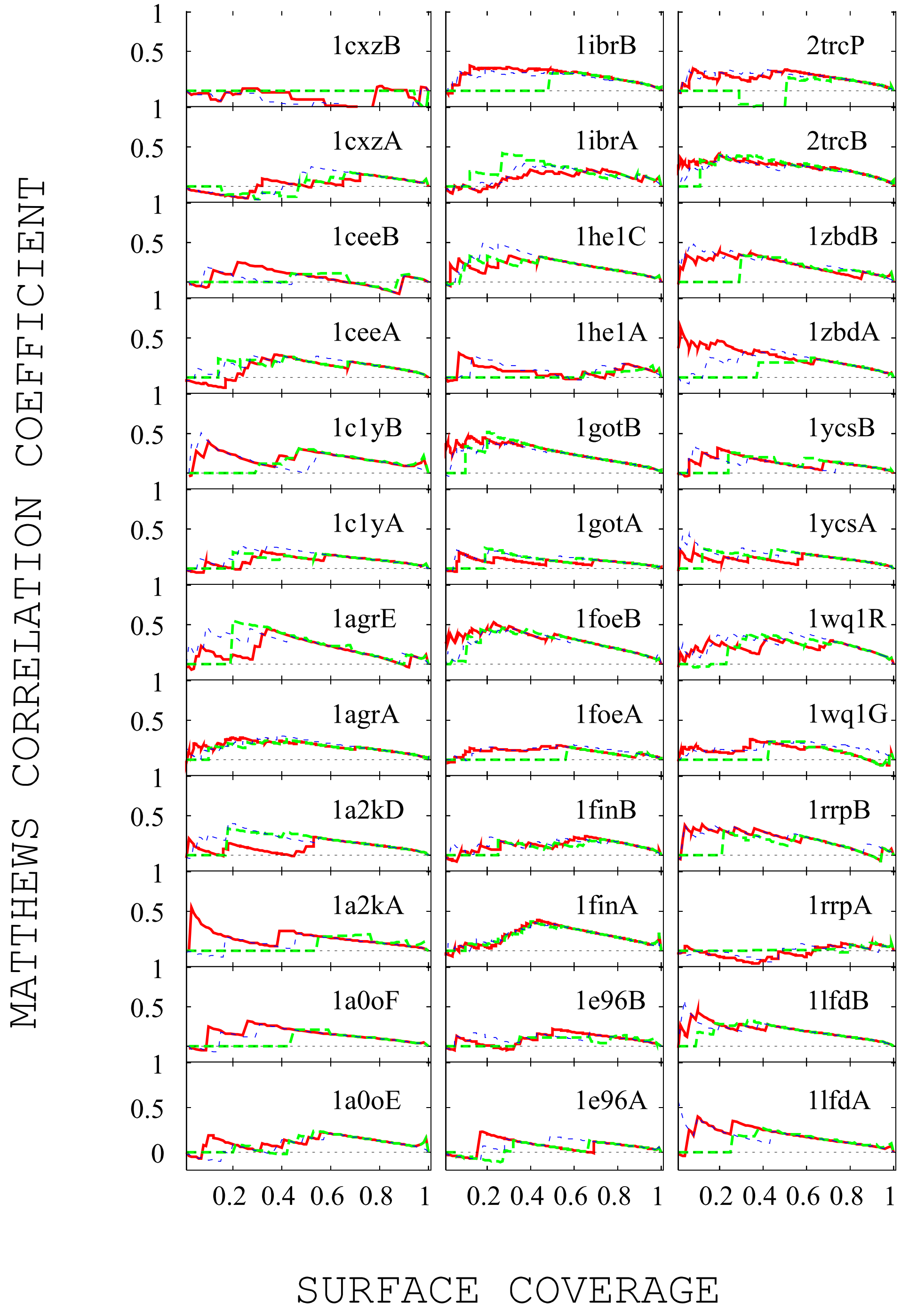

Figure 1: Matthews coefficient as a function of surface coverage, using the same sequence sets as in Fig. 3 in the main text. Red: H_{BB}; green: column entropy; blue: rate4site.

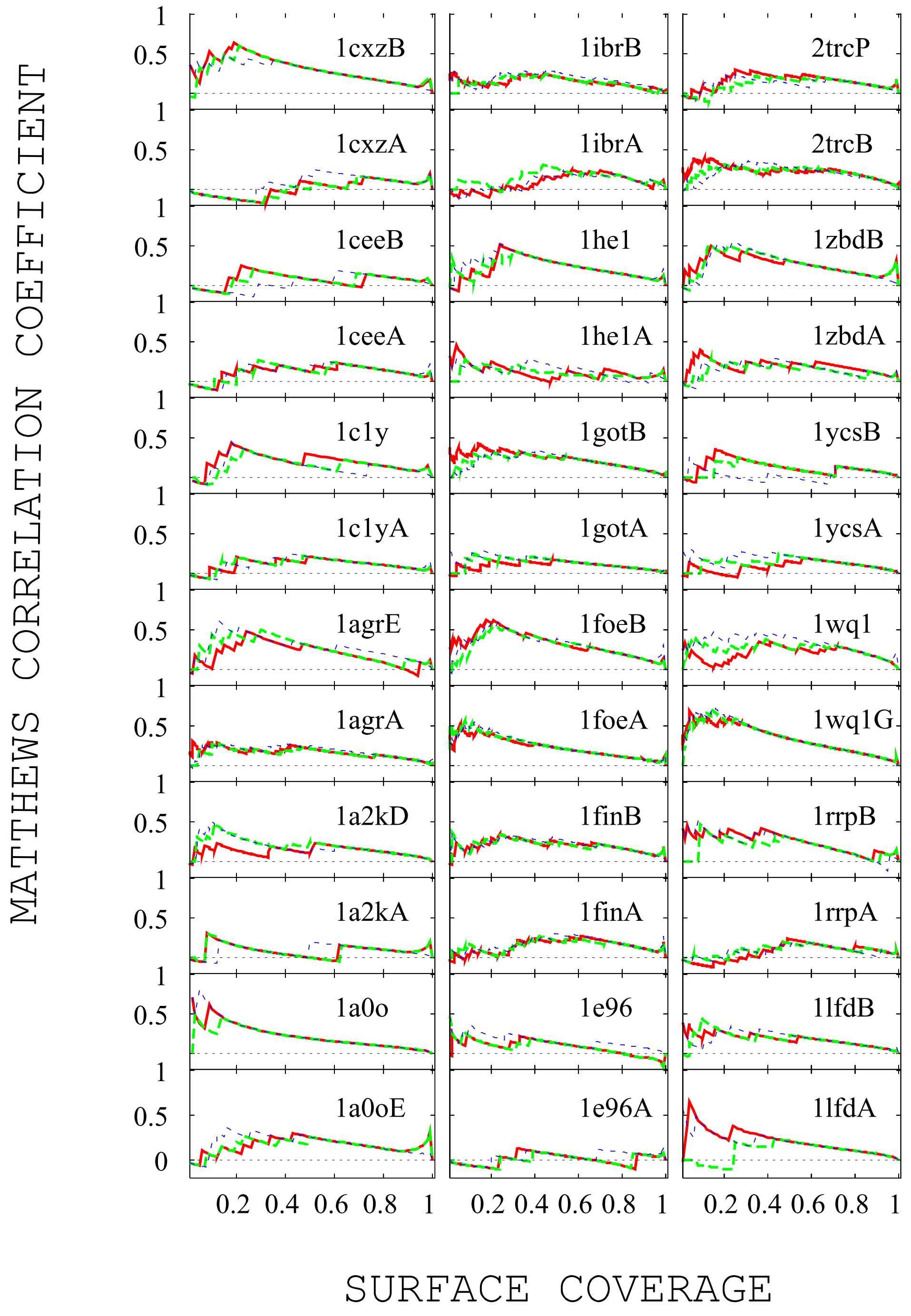

Figure 2: Matthews coefficient as a function of surface coverage, using the same sequence sets as in Fig. 4 in the main text.Red: H_{BB}; green: column entropy; blue: rate4site.

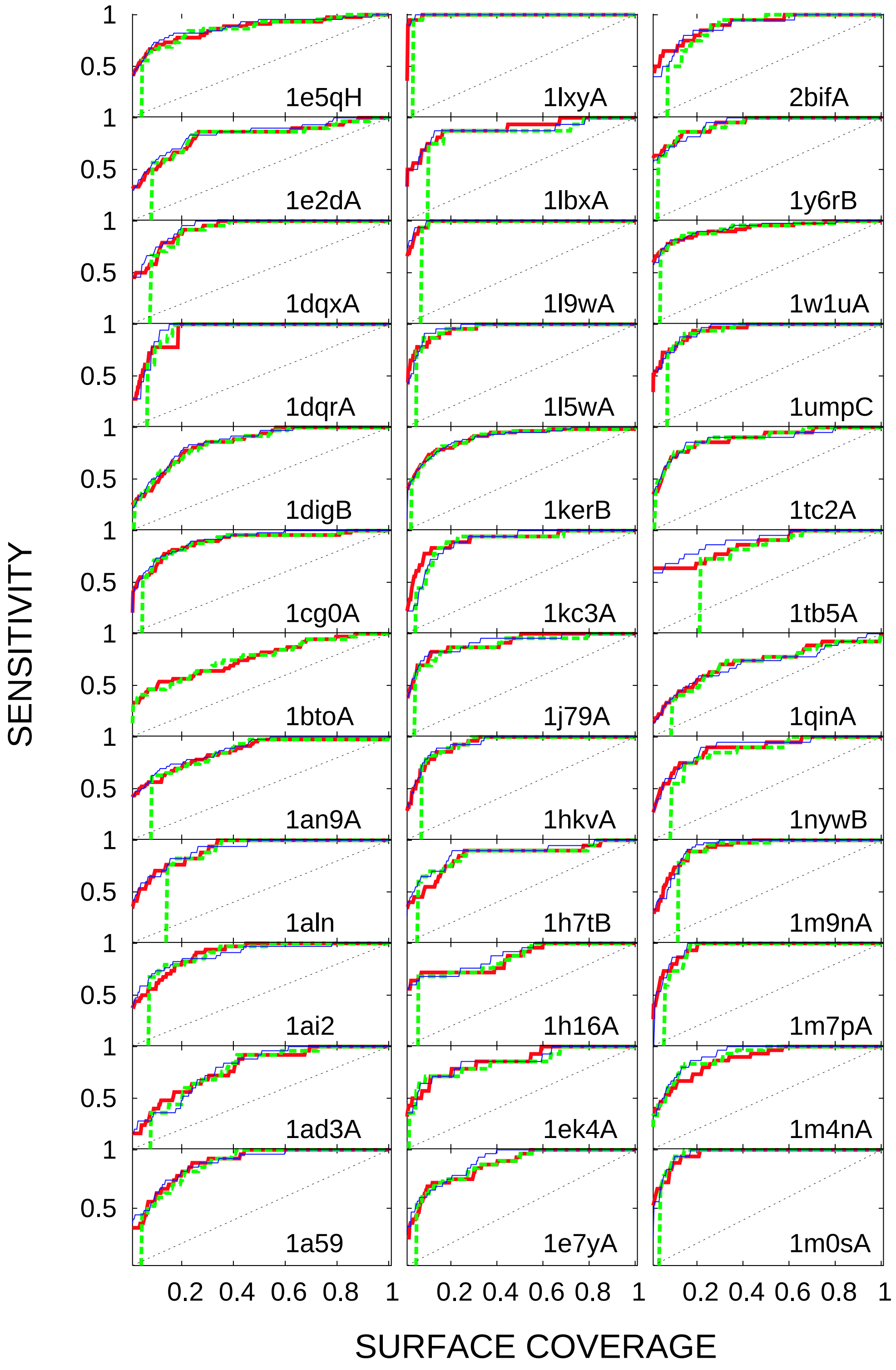

Figure 3: The ability of three different methods to detect catalytic sites of enzymes. The functional site is defined here as the set of residues within 5$\mathring{A}$ of the substrate or the cofactor. Horizontal axis: fraction of surface appearing among the top scoring residues (surface coverage). Vertical axis: fraction of catalytic site detected. Thick full line: Kullback-Leibler joint entropy; thick dashed line: column entropy; thin line: rate4site. (The alignment used for 1btoA was too large for rate4site). Protein Databank Identifier of each protein is indicated in the corner of each panel. The sequences are selected as in Fig. 4 in the text.

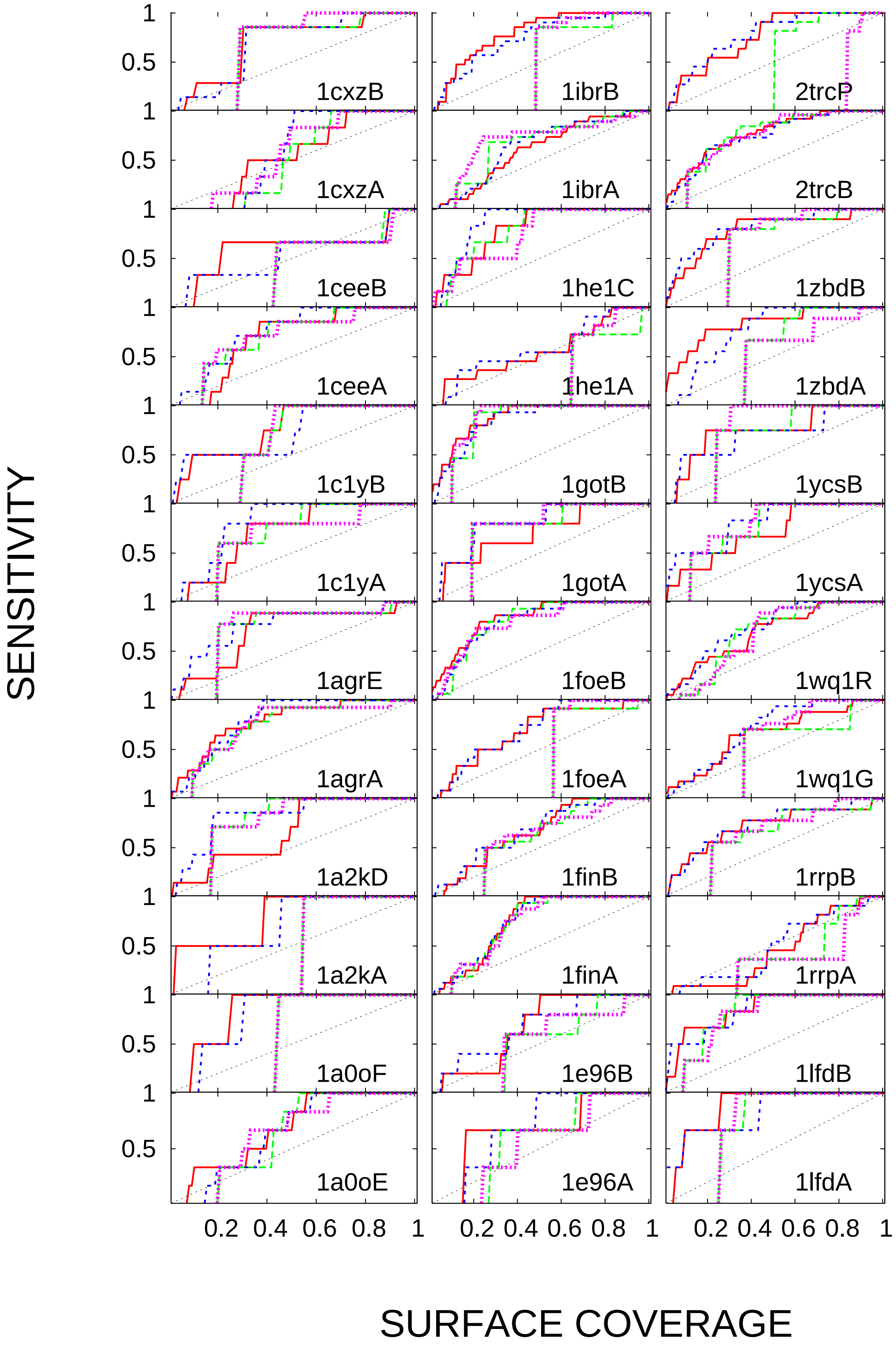

SENSITIVITY
SURFACE COVERAGE
1cxzB
1ibrB
2trcP
1cxzA
1ibrA
2trcB
1ceeB
1he1C
1zbdB
1ceeA
1he1A
1zbdA
1c1yB
1gotB
1ycsB
1c1yA
1gotA
1ycsA
1agrE
1foeB
1wq1R
1agrA
1foeA
1wq1G
1a2kD
1finB
1rrpB
1a2kA
1finA
1rrpA
1a0oF
1e96B
1lfdB
1a0oE
1e96A
1lfdA

Figure 4:

Figure 4 (Previous page): Comparison of the method of Valdar and Thornton[1,2] with the methods used in the main text. The test set is the same as in Fig. 3 in the main text. Full red line: joint entropy, evaluated according to Eq.6 in the text; dashed green line: Shannon entropy; dashed blue line: rate4site. Pink: Valdar. The results obtained using the method described in the text (H_{BB}) differ from Valdar's with p-value of 1×10^{-4} on the Wilcoxon test. Valdar's method was designed to deal with unequal sampling from the sequence space, not a very strong issue in a set of close homologues used here. By setting the diagonal elements in the scoring matrix to the same average value ([1]), the method starts suffering from the same problem as the entropy - namely the inability to distinguish among different conserved amino acid types.

References

[1] Valdar W, Thornton J: **Protein–protein interfaces: Analysis of amino acid conservation in homodimers**. *Proteins Structure Function and Genetics* 2001, **42**:108–124.

[2] Valdar W: **Scoring Residue Conservation**. *Proteins Struct. Funct. Genet.* 2002, **48**:227–241. [Http://www.ebi.ac.uk/thornton-srv/databases/cgi-bin/valdar/scorecons_server.pl].

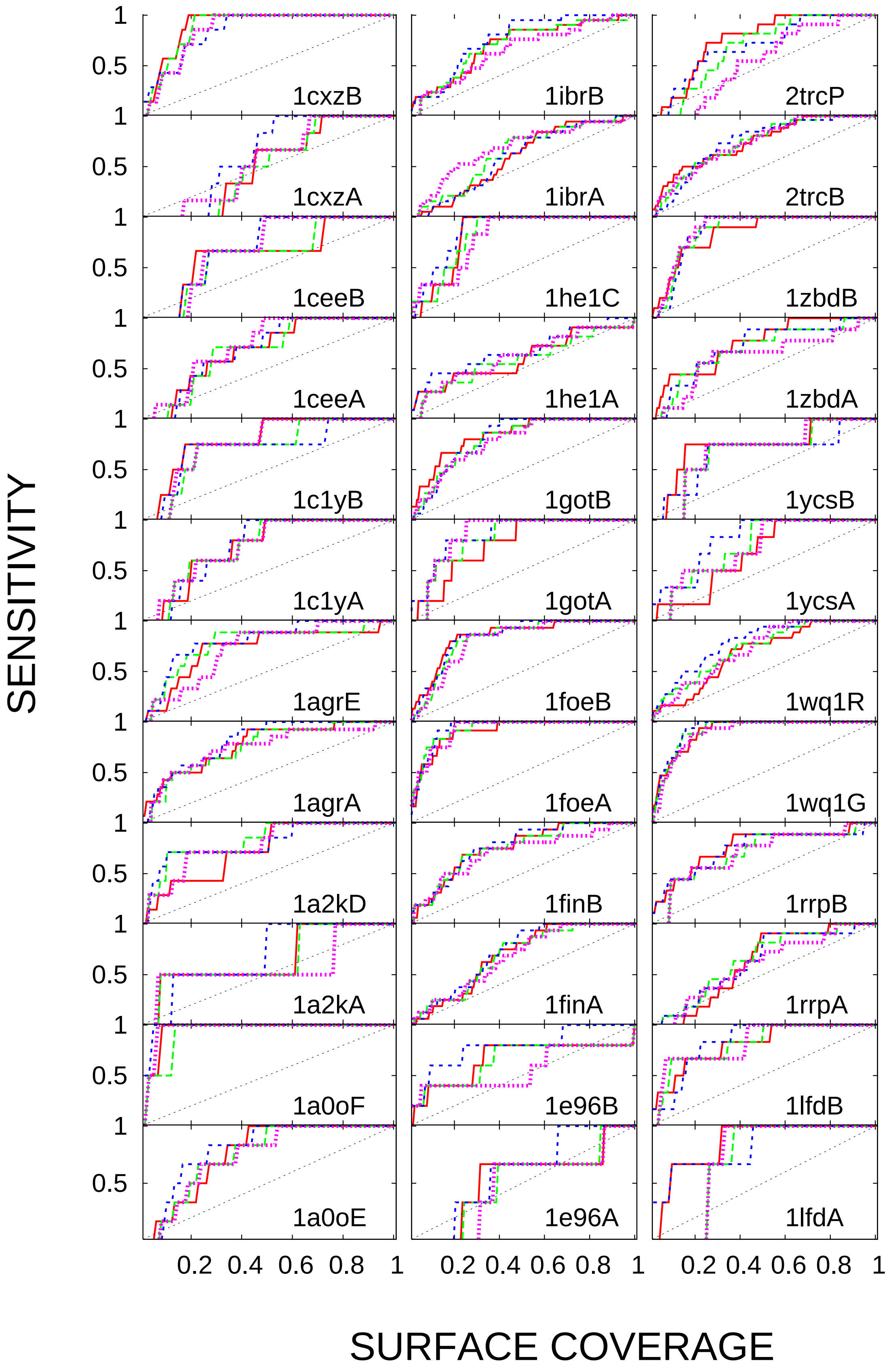

Figure 5: The same as Fig. 4 in this Supplement, using the set of more distant homologues (the same as Fig. 4 in the main test). The differences in performances of different methods are not statistically significant.
